# Systematic depression screening in high-risk patients attending primary care: a pragmatic cluster-randomized trial

**DOI:** 10.1186/1471-244X-13-83

**Published:** 2013-03-13

**Authors:** Irene Romera, Ángel L Montejo, Enric Aragonés, José Ángel Arbesú, Celso Iglesias-García, Silvia López, José Antonio Lozano, Sireesha Pamulapati, Belen Yruretagoyena, Inmaculada Gilaberte

**Affiliations:** 1 Clinical Research Department, Lilly, S.A., Avenida de la Industria, 30. Alcobendas, E-28108, Madrid, Spain; 2 University Hospital of Salamanca. IBSAL. Department of Psychiatry, University of Salamanca, Salamanca, Spain; 3 Centro de Atención Primaria Constantí, Tarragona, Spain; 4 Centro de Salud de la Eria, Oviedo, Spain; 5 Hospital Valle del Nalón, Centro de Salud Mental de Langreo, Langreo, Spain; 6 Atención Primaria, Área 7 de Madrid, Madrid, Spain; 7 Centro Jóse Antonio Lozano, Sevilla, Spain; 8 European Statistics, Lilly UK, Erl Wood Manor, Windlesham, Surrey, United Kingdom; 9 Universitat Autonoma de Barcelona, Barcelona, Spain

**Keywords:** Major depressive disorder, Guideline adherence, Diagnosis, Primary health care, Screening

## Abstract

**Background:**

Systematic screening for depression in high-risk patients is recommended but remains controversial. The aim of this study was to assess the effectiveness of such screening in everyday clinical practice on depression recognition.

**Methods:**

A pragmatic, cluster randomized, controlled study that randomized primary care physicians (PCPs) in Spain either to an intervention or control group. The intervention group (35-PCPs) received training in depression screening and used depression screening routinely for at least 6 months. The control group (34-PCPs) managed depression in their usual manner. Adherence to (1–6; never-very frequently), feasibility (1–4; unfeasible-very feasible), and acceptance (1–5; very poor-very good) of the screening were evaluated. Underrecognition (primary outcome) and undertreatment rates of major depressive disorder (MDD) in the two groups were compared 6 months after randomization in a random sample of 3737 patients assigned to these PCPs using logistic regression adjusting for the clustering effect.

**Results:**

No significant differences were found for recognition rates (58.0% vs. 48.1% intervention vs. control; OR [95%CI] 1.40 [0.73-2.68], p = 0.309). The undertreatment rate did not differ significantly either (p = 0.390). The mean adherence to depression screening was 4.4 ± 1.0 (‘occasionally’), the mean feasibility was 3.1 ± 0.5 (‘moderately feasible’), and the mean acceptance was 4.2 ± 0.6 (‘good’).

**Conclusions:**

This research was not able to show effectiveness of the systematic screening for MDD in high-risk patients on depression recognition in primary care. The poor adherence to screening implementation could partially explain the results. These reflect the difficulties of putting into practice the clinical guidelines usually based on interventional research.

**Trial registration:**

Clinicaltrials.gov NCT01662817

## Background

Major depressive disorder (MDD) is a common disease associated with significant disability and high healthcare costs, but it is still underdiagnosed in a large percentage of primary health care (PC) patients [1-3]. Not recognizing depression may prolong episodes, increasing the duration of patient suffering and decreasing the likelihood of recovery [4]. Systematic screening for depression has therefore been recommended in order to reduce the large rate of depression underrecognition and its deleterious consequences [5-8].

Evidence from explanatory clinical trials, indicates that screening ensures proper identification of depressed patients in the primary health care setting, and several health agencies have published guidelines recommending systematic screening for depression in high-risk patients [5-8]; however, when incorporated into routine primary care practice, their effectiveness is controversial [9-12].

The guidelines on systematic screening for depression recommend, as one possible screening strategy, asking the patient two questions about their mood and anhedonia, followed by a diagnostic interview if screening is positive [5-8]. More specifically, it has been proposed by the US preventive Services Task Force, that depression screening in primary care should be aimed at patients at high risk of depression, such as patients with a history of depression, psychological comorbidities, unexplained somatic symptoms, or disability due to physical illnesses [13]. It has been claimed that screening in PC ensures that depressed patients are identified [5-8], but evidence is based on explanatory trials performed under the inherent “ideal” conditions of clinical trials [7,14,15]. In fact, there is a lack of randomized data supporting their effectiveness when integrated into the everyday clinical practice. The authors have identified only one non-randomized study that aimed to assess the effectiveness of depression screening under such conditions [16]. In this study, Bass et al., evaluated the effectiveness of screening on treatment initiation in three high-risk groups in primary care (patients with mental health problems, unexplained somatic complaints and patients who frequently consult their general practitioner). Negative results were reported [16]. Two further studies have been published assessing the extent of implementation of screening in the PC setting [17,18], showing very low [17] and very high [18] use of depression screening. This lack of effectiveness data could partly explain the controversy of systematic screening for depression in PC.

The present paper presents the results of a pragmatic research that tested the hypothesis whether implementing the guidelines on screening for depression in high-risk patients in everyday clinical practice reduces the underrecognition of MDD in PC. The authors also addressed MDD undertreatment and outcomes as secondary endpoints and sought to evaluate the degree of adherence and acceptance of depression screening by primary care physicians (PCPs).

## Methods

### Study design

This was a pragmatic, cluster-randomized, controlled study performed in PC practices with the PCP as the unit of randomization (cluster). One hundred five PCPs from the public healthcare system throughout Spain were invited by telephone between July and September of 2009. Sixty-nine (66%) PCPs, who fulfilled the inclusion criteria, were randomized. Randomization of PCPs to either an intervention group (n = 35) or a control group (n = 34) was stratified by the number of patients attending their practices daily (<50, ≥50) and by PCP shift (morning or afternoon). Participating PC practices belong to the Spanish primary health system that offers almost universal coverage, provides free access and partial reimbursement for the majority of antidepressant treatments [19]. The organization of the system is regionally controlled by 17 autonomous communities. The pay of PCPs is not linked to integrated care or management of disease [19].

**Figure 1:**
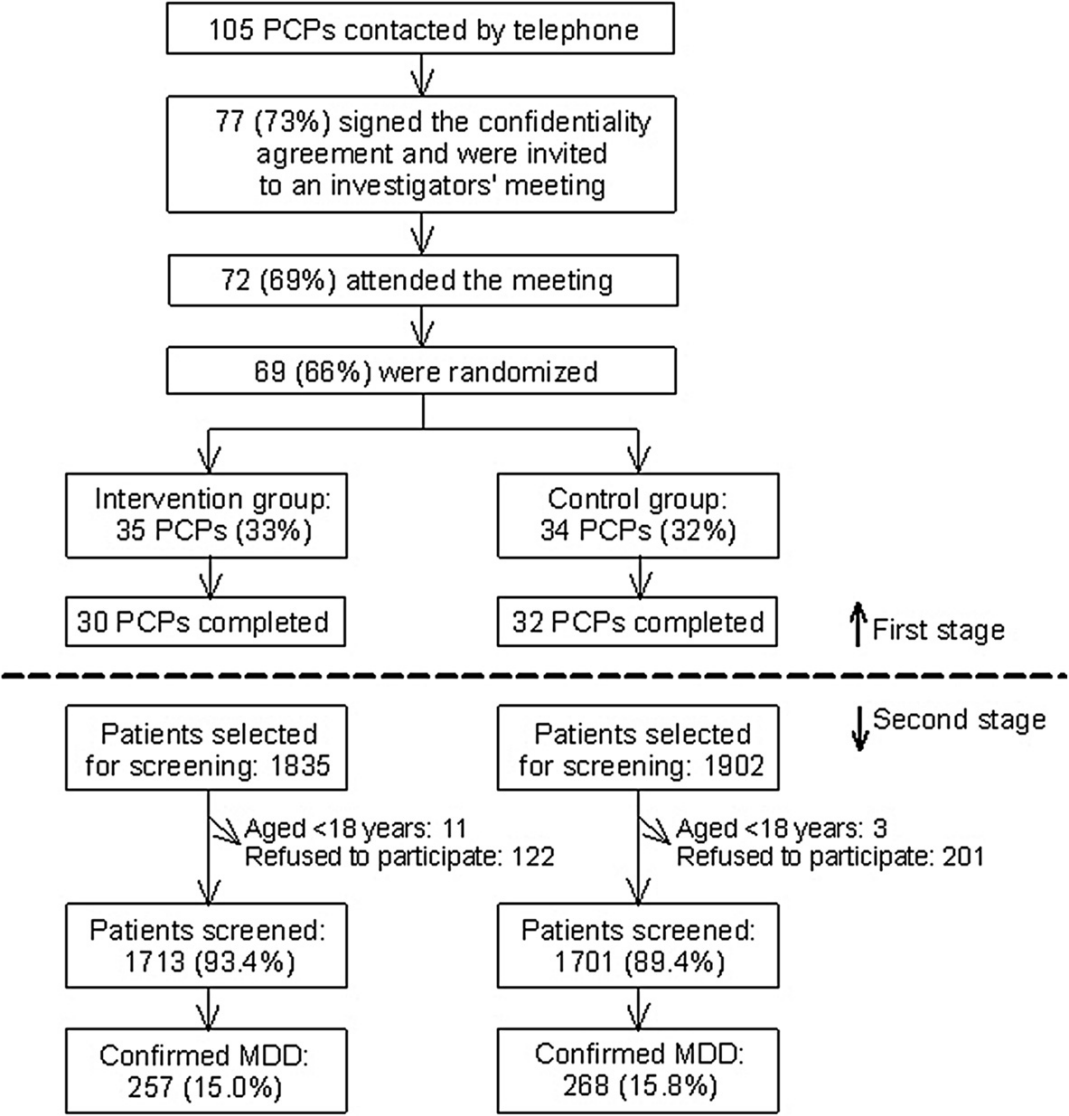
**Study flow chart.** PCP: primary care physician. MDD: major depressive disorder. First stage: PCP randomization (September 2009) and implementation of the intervention (October 2009 to March 2010). Second stage: collection of patient data (April to July 2010). Selection of patients for screening in the second stage: Every 10th attending patient was evaluated (n = 3737) by the PCP with the Depression subscale of the Hospital Anxiety and Depression Scale. Confirmation of MDD in the study sample was done with the Mini-International Neuropsychiatric Interview.

In a second stage, at least six months after randomization, the effectiveness of the intervention was cross-sectionally evaluated at patient level between April and July 2010 (Figure 1) by the same PCPs. Following the next procedure, a sample of patients with MDD episode (n = 525) was systematically recruited (Figure 1). Every 10th attending patient was evaluated (n = 3737) by the PCP with the Depression subscale of the Hospital Anxiety and Depression Scale (HADS-D) [20]. The Hospital Anxiety and Depression Scale was designed to screen for the presence of a mood disorder in medically ill patients. The HADS-D is a self-report scale and contains 7 items rated on 4-point Likert-type scales [20]. The sensitivity and specificity for the HADS-D is approximately 0.80 [21]. Patients with a HADS-D score of 8 or higher were then interviewed by the PCP with the Mini-International Neuropsychiatric Interview (MINI) [22] to confirm a MDD episode (n = 525) (Figure 1). The MINI is an abbreviated psychiatric structured interview used to diagnose major Axis I psychiatric disorders in DSM-IV and ICD-10. The MINI is a relatively brief instrument that is divided into modules corresponding to diagnostic categories such as major depressive episode, dysthymia, mania/hypomania, panic disorder, etc. The module administrated was that corresponding to the diagnostic category for MDD [22].

The study was carried out between September 2009 and July 2010 (PCP randomization – September 2009; implementation of the intervention – from October 2009 to March 2010; and collection of patient data, second stage – April to July 2010). It was approved by the ethical review board of Hospital Clínico Universitario San Carlos, Madrid and carried out according to the principles of the Declaration of Helsinki [23] and the Spanish regulations (circular 15/2002/AEM).

### Selection criteria

PCPs were fully informed of the study design and agreed to be randomized to the intervention or control group. PCPs were excluded if they already followed the recommendations on screening or were planning to do so, if they would be absent from their practice for a significant period during the study, or if they could not ensure effective management of depression (diagnosis, treatment and follow-up is not ensured).

In the second stage, patients were included if they were aged 18 years or older, and provided written consent for the collection and use of their clinical data collected by the PCP. High-risk patient and non-high-risk patients were suitable for this second stage. Patients not able to understand the aims of the study based on PCP clinical criteria were excluded. The study population was formed by the patients with a MDD episode according to the MINI.

### Intervention

PCPs randomized to the intervention arm received a 1-day face-to-face training, done by one psychiatrist and four PCPs, on the recommendations on screening for depression in adults, according to the U.S. Preventive Services Task Force (USPSTF) 2002 guidelines [7,13], and were asked to implement them in their routine clinical practice for at least 6 months. Monthly reminders were sent by e-mail. They were also required to complete a form each month indicating adherence to and acceptance and feasibility of such recommendations.

The 2002 USPSTF guidelines recommended, as a way of conducting the screening, to ask the patient the following two questions: “Over the past two weeks, have you felt down, depressed, or hopeless?” and “Over the past two weeks, have you felt little interest or pleasure in doing things?”. “High risk” was defined as fulfilling at least one of the following: history of depression, somatic symptoms without any cause, psychological comorbidities or drug abuse, or chronic pain. Positive screening should be followed by a diagnostic interview. The training included several workshops with clinical cases to discuss the barriers of implementation.

### Outcome measures

The primary outcome measure was the rate of underrecognized depression, measured in the second stage, six months after the PCPs were randomized. Underrecognition was assessed by a systematic review of the patients’ medical records carried out by the participating physicians. Patients with a confirmed MDD episode were considered as underrecognized if there was not any diagnosis of such episode in the patient’s medical record, regardless the patient was receiving or not any treatment. There was not any specific time-frame (for example, last 6 months) for the revision of the medical record.

For the secondary outcome measure of undertreatment, patients with a confirmed MDD episode were considered “treated “ if at the time of the assessment, they were receiving any antidepressant medication, any non-pharmacological treatment for depression according to the NICE guidelines [5], or if they had been referred to specialized psychiatric care for the current episode. NICE guideline was chosen since it is a complete and well-known guide by physicians in Spain.

The feasibility of implementing the depression screening by the PCPs randomized to the intervention group as well as its acceptance and degree of adherence were measured monthly using Likert-type scales developed specifically for this study. Feasibility was scored from 1 (unfeasible) to 4 (very feasible), acceptance from 1 (very poor) to 5 (very good), and adherence from 1 (never) to 6 (very frequently). In addition, the average number of patients in whom the screening was applied was collected monthly by the PCPs.

To assess depression outcomes, data on severity of depression was collected using the Clinical Global Impression-Severity scale (CGI-S), which ranges from 1 (normal, no disease) to 7 (severely diseased) [24]. Functioning was evaluated by the Sheehan Disability Scale (SDS), a self-administered questionnaire which evaluates changes in the subjects’ working, social, and family life [25]. Each area is scored between 0 (no perturbation) and 10 (maximum perturbation), up to a total score of 30 for the whole scale [25]. In addition, work absenteeism due to depression, median duration of the current MDD episode and reason for consultation (multiple choice between emotional/affective symptoms, somatic/physical symptoms, social reasons, requires medication/prescription or other) for the study visit were also collected. These depression outcomes were measured in the second stage, at least six months after PCPs randomization, and were collected by the PCPs.

### Sample size calculation

Sample size was calculated assuming that the rate of depression underrecognition in the PC setting is about 50% [26] and that intervention reduces it by 15% [7]. Considering that randomization was done at PCP level and each practice was expected to recruit 5 patients with a 0.05 intra-cluster correlation [27], 432 patients with a MDD episode were needed. With a prevalence of MDD of 14% in PC [26] and an 80% sensitivity of the HADS-D scale [27], about 4000 patients were to be screened.

### Statistical methods

Underrecognition rates were compared between groups by logistic regression modelling adjusted for the effect of cluster aggregation by using generalized estimating equations. For each patient, the dependent variable was the presence or absence of MDD diagnosis in the medical records. Independent variables on the PCP level were the number of patients attending the practice daily (50 or more vs. less than 50 patients) and the PCP’s shift (morning vs. afternoon), self-reported by the PCP. PCP’s shift was included since could has an effect on the primary outcome (patients in the afternoon shift are usually younger and have less co-morbidities than patients in the morning shift) as well as the number of patients attending the practice daily (more patients could be linked with more under-recognition of depression). On the patient level, the independent variables were gender, age (continuous variable), education (no formal education vs. primary education; vs. vocational training; vs. secondary education; vs. university education), work status (unable to work vs. unemployed; vs. house-keeping; vs. retired; vs. working for pay), medical comorbidities (yes vs. no), psychiatric comorbidities (yes vs. no), non**-**psychiatric treatment (yes vs. no), and time since previous visit (continuous variable). A similar model including the same covariates was used to analyze the differences between groups in the undertreatment of depression.

The duration and severity of depression, the patient’s functioning, and the number of days absent from work due to depression, were analyzed using an extension of covariance analysis. The model was adjusted for the grouping effect caused by the clustering, with the practice considered as a random effect. The analysis was also adjusted by the same covariates described in the models above and both, pharmacological treatment for depression (yes vs. no) and non-pharmacological treatment for depression (yes vs. no). The reason for consultation (emotional/affective symptoms yes vs. no; somatic/physical symptoms yes vs. no) was included in a post hoc analysis as an independent variable in the logistic regression model for underrecognition of depression.

All variables were described using descriptive statistics. All statistical tests were two-tailed with a level of significance of p = 0.05, unless otherwise specified.

SAS (Statistical Analysis System) version 9.2 was used for analysis.

## Results

### Physicians, primary care practices and patients

The characteristics of the PC practices and PCPs are shown in Table 1. Demographic and clinical characteristic of the patients are shown in Table 2. Before study visit, most patients (436/525; 83%) had visited their PCP (84.8% and 81.3%; intervention and control groups respectively), with a mean of 3.4 ± 2.70 visits in the previous 3 months.

**Table 1:** Characteristics of the primary care practices and physicians participating in the study

	**Intervention group N = 35**	**Control group N = 34**
**Primary care practices**		
City population (thousands), mean (range)	287.6 (1.4-3213.3)	887.9 (1.6-3213.3)
Patients attending practice daily (<50), n (%)	24 (68.6)	24 (70.6)
Geographical area, n (%)		
Central Spain	11 (31.4)	14 (41.2)
Mediterranean coast	13 (37.1)	7 (20.6)
North of Spain	3 (8.6)	6 (17.6)
South of Spain	8 (22.9)	7 (20.6)
**Primary care physicians**		
Gender (male), n (%)	23 (65.7)	28 (82.4)
Shift (morning), n (%)	31 (88.6)	30 (88.2)

**Table 2:** Characteristics of the primary care patients participating in the study

	**Intervention group N = 257**	**Control group N = 268**
Female, n (%)	191 (74.3)	197 (73.5)
Mean age (SD)	54.8 (16.1)	56.2 (16.3)
Marital status, n (%)		
Married/Partnered	149 (57.9)	160 (59.7)
Divorced/separated	21 (8.2)	17 (6.3)
Widowed	30 (11.7)	41 (15.3)
Other	57 (22.2)	50 (18.7)
Educational status, n (%)		
Primary education	107 (41.6)	111 (41.4)
Secondary education	46 (17.9)	40 (14.9)
University	21 (8.2)	37 (13.8)
Other	83 (32.3)	80 (29.8)
Any medical co-morbidity*, n (%)	167 (65.0)	185 (69.0)
Any psychiatric co-morbidity**, n (%)	142 (55.3)	160 (59.7)

* Hypertension, Osteoarthritis, Hyperlipidemia, Obesity, Hypercholesterolemia, Diabetes Mellitus, Migraine, Fibromyalgia Syndrome, Carcinoma, Cardiovascular Disease, Pulmonary Disease, Cardiac Arrest, Myocardial Infarction, Cerebrovascular Disease, Parkinson’s Disease.

** Dysthymia, adjustment disorder, anxiety, anorexia, phobic disorder, panic disorder, alcohol abuse, bulimia nervosa, post traumatic stress disorder, drug dependence, bipolar disorder, psychotic disorder, obsessive-compulsive disorder.

### Underrecognition of depression

No significant differences were found in the rate of underrecognition of depression between intervention and control groups (underrecognition rates were 33.9% vs. 41.4% intervention vs. control; recognition rates were 58.0% vs. 48.1% intervention vs. control; OR [95% CI] for depression recognition intervention vs. control: 1.40 [0.73-2.68], p = 0.309) (Table 3). The factors related to the PCP did not yield significant differences either. Two patient-related factors significantly improved recognition rate: psychiatric comorbidities (OR [95% CI]: 2.43[1.44-4.08], *p* < 0.001 vs. absence of psychiatric comorbidities) and inability to work (OR [95% CI]: 2.91[1.22-6.95], *p* = 0.016 vs. working for pay).

**Table 3:** Recognition and treatment rates of depression

	**Intervention group N = 257**	**Control group N = 268**	**OR**	**95% CI for OR**	***p*-value**
Recognized patients, n (%)	149 (58.0)	129 (48.1)	1.40	0.73-2.68	0.309
Underrecognized patients, n (%)	87 (33.9)	111 (41.4)			
Missing data, n (%)	21 (8.2)	28 (10.4%)			
Treated patients, n (%)	153 (59.5)	139 (51.9%)	1.35	0.68-2.63	0.390
Undertreated patients, n (%)	104 (40.5)	129 (48.1)			

OR: odds ratio. CI: confidence interval.

### Undertreatment of depression

No significant differences were found between study groups (Table 3). Factors associated with less undertreatment were the presence of psychiatric comorbidities (OR [95% CI]: 0.25 [0.15-0.40], *p* < 0.001 vs. absence of psychiatric comorbidities) and inability to work (OR [95% CI]: 0.29 [0.12-0.71], *p* = 0.007 vs. working for pay). Significantly more patients without formal education were undertreated than those with education (OR [95% CI]: 2.26 [1.18-4.33]), *p* = 0.014 vs. primary education; OR [95% CI]: 3.80 [1.48-9.77], *p* = 0.006 vs. secondary education; OR [95% CI]: 2.92 [1.10-7.75] *p* = 0.031, vs. university education).

### Implementation of the screening

Five out of 35 PCPs randomized to the intervention group dropped out from the study (Figure 1). In the 30 PCPs that continued in the study, the mean (± standard deviation) adherence score for the whole study period was 4.4 ± 1.0 (“occasionally”). Between 40% (Month 1) and 60% (Month 3) of PCPs followed the recommendations “frequently” or “very frequently”. Overall, PCPs used the screening in an average of 67 patients (95% CI: 38.1-96.5) per month.

The mean score for feasibility of implementation was 3.1 ± 0.5 (“moderately feasible”). Nearly 90% of PCPs found the implementation “moderately feasible” or “very feasible” at all monthly assessments.

The mean score for acceptance of the screening among PCPs was 4.2 ± 0.6 (“good”). Most PCPs (90%) assessed acceptability as “good” or “very good” (Table 4).

**Table 4:** Implementation degree of the recommendations among the primary care physicians randomized to the intervention group

	**Month 1 N = 30**	**Month 2 N = 30**	**Month 3 N = 30**	**Month 4 N = 30**	**Month 5 N = 30**	**Month 6 N = 29**
**Adherence, n (%)**						
Very frequently/ Frequently	12 (40.0)	13 (43.3)	18 (60.0)	16 (53.3)	15 (50.0)	15 (51.7)
Occasionally	11 (36.7)	9 (30.0)	5 (16.7)	10 (33.3)	10 (33.3)	9 (31.0)
Rarely/very rarely/Never	7 (23.3)	8 (26.7)	7 (23.3)	4 (13.3)	5 (16.7)	5 (17.2)
**Feasibility, n (%)**						
Very feasible	4 (13.3)	6 (20.0)	9 (30.0)	9 (30.0)	9 (30.0)	8 (27.6)
Moderately feasible	23 (76.7)	19 (63.3)	17 (56.7)	18 (60.0)	17 (56.7)	18 (62.1)
Of little feasibility/ Unfeasible	3 (10.0)	5 (16.7)	4 (13.3)	3 (10.0)	4 (13.3)	3 (10.3)
**Acceptance, n (%)**						
Very good/Good	27 (90.0)	26 (86.7)	27 (90.0)	27 (90.0)	27 (90.0)	26 (89.7)
Barely acceptable	2 (6.7)	3 (10.0)	2 (6.7)	3 (10.0)	3 (10.0)	3 (10.3)
Poor/Very poor	1 (3.3)	1 (3.3)	1 (3.3)	0	0	0

### Depression outcomes

No significant differences were found between patients in the intervention and control groups for severity of depression (CGI-S), functional impairment (SDS), mean duration of the episode, or mean days on sick leave. Overall, patients had a mean (SD) CGI-S score of 4.1 ± 0.81 and a mean (SD) SDS score of 18.3 ± 6.0, corresponding to a moderate degree of depression and functional impairment. The mean (SD) duration of the current MDD episode was 408 ± 1288 days; 12% of patients were on sick leave due to depression, with a mean (SD) of 159 ± 477 days.

### Reasons for consultation

The most frequent reason for consultation in patients with a non-recognized MDD episode was somatic-physical symptoms (107/198 patients, 54%). In contrast, it was emotional-affective symptoms (136/278 patients, 49%) in patients with a recognized MDD episode. Consultation for somatic symptoms was associated with lower recognition rates (OR [95% CI]: 0.58 [0.37-0.90], *p* < 0.016 vs. no somatic symptoms), while consultation for emotional symptoms was associated with a higher recognition rate (OR [95% CI]: 2.74 [1.59-4.72], *p* < 0.001 vs. no emotional symptoms).

## Discussion

### Summary of main findings

Contrary to our hypothesis, we did not find significant differences for the underrecognition rate of MDD between patients treated by PCPs who followed guidelines for screening and those treated by PCPs who did not. Although the guidelines found high acceptance among PCPs in the intervention group, adherence to them was suboptimal. Our results also failed to show a significant difference between the study groups regarding depression undertreatment.

### Comparison with existing literature

We did not find a statistically significant difference of MDD underrecognition rates between study groups. This negative result contrasts somewhat with the efficacy of screening guidelines reported in controlled clinical trials [7,8,15]. The failure to detect differences in the secondary outcome of depression undertreatment is, nevertheless, consistent with prior reports that point to the ineffectiveness of screening alone to resolve this issue in PC [14,16].

With regard to underrecognition of depression, a study done by Caballero et al. [26] carried out in PC setting in Spain, that used a similar design to our study, reported that 54% of PC patients with MDD in Spain remain unrecognized. However, in our study we found that only 41% of control group patients remained unrecognized. This may have contributed to reduce between-group differences in this study.

Adherence to the screening among the PCPs in the intervention group was suboptimal: only about half of them used the screening in their practice frequently or very frequently during the study. In contrast, 90% of PCPs considered that its acceptance was good or very good. Our adherence data are better than the results reported by Harrinson et al. [17], however, worse than Kirkaldy et al. [18]. A study conducted by Harrison et al. [17] in primary care centers through the United States showed that, despite the recommendations raised by the USPSTF, only the 2.3% of the physicians performed the screening for depression. Kirkaldy et al. evaluated the screening program implemented in a medical center for one month and found that the 97% of PCPs performed the screening [18]. Our results on acceptance and adherence suggest that many PCPs were willing to implement the screening recommendations but were unable to do that frequently. PCPs in our study setting have a very short consultation time with each patient, they have no support staff for depression care and are not audited and paid for screening [19]. Besides, PCPs have other conflicting clinical priorities [19,28]. These factors may prevent physicians from establishing new systems, even if acceptable and feasible [29]. The suboptimal adherence found in our study is in accordance with a previous study where PCPs had a positive view of the NICE guideline for depression, but its impact was compromised by resource and practitioner barriers to implementation [30]. The degree of implementation of new interventions into routine clinical practice is markedly influenced by contextual factors in the setting where they have to be applied [31].In order to improve the quality care for depression, organizational barriers to the implementation of a depression guideline, in the complex realities of PC, is a significant factor to take into account. In that sense, it should be mentioned that collaborative care in primary care has shown effectiveness in the management of depression [32,33]. However, this is a multi-component approach which requires the involvement of additional staff like nurses. Currently, it is not a usual practice for the management of depression in Spain [33]. The aim of this study was to test the implementation of a specific recommendation regarding depression screening in the current everyday reality of the primary care setting in Spain.

The present study was based on the 2002 USPSTF recommendations [7,13]. These recommendations are in accordance with EU guidelines that are known and used in Spain [5], and with Canadian guidelines [5,6]. However, we chose the USPSTF guidelines because they were more detailed and had more information regarding their implementation. The version issued in 2002 recommended screening only in those cases where the patient could have adequate follow-up and treatment in case depression was recognized. The 2009 recommendations are more restrictive and specify that the screening should not be applied unless there is support from additional staff to provide depression care [8]. PCPs in our study lack additional staff, and as previously discussed, this could in part explain the suboptimal implementation of the recommendations by some PCPs randomized to the intervention group, and therefore the study results.

Consistent with early research, somatic symptoms were a frequent reason for consultation and were associated with underdiagnosis of depression [34]. This finding supports the additional vigilance in patients with somatic symptoms. Also, PCPs should be aware that, in patients with medical illnesses, an interview focussed on the affective and cognitive symptoms of depression could be helpful and easier to apply [35].

Finally, psychiatric comorbidities and inability to work were associated with recognition. Maybe these conditions can sound an alarm to the PCP, so he/she is more driven to look for other diagnosis.

### Strengths and limitations of the study

Because of the pragmatic, cluster-randomized design, this study allowed to assess the effectiveness rather than the efficacy (under controlled conditions) of depression screening. This may be construed as a strength because the evidence obtained under everyday practice conditions serves as a basis for decisions about healthcare policies [36]. To evaluate the recognition of the depression episode, the participating PCPs reviewed the patients’ medical records themselves, so this may have introduced a collection bias, so a possible deflation of underrecognition rates cannot be ruled out. However, this procedure was done in both study groups. The use of an independent evaluator would have been optimal. However, this option was ruled out after the evaluation of the pros and cons, taking into account the difficulties of its implementation in the 69 participating centres. There was not any specific time-frame for the revision of the medical record. This adds complexity to the collection of information and could be considered a limitation. However, it allows the capture of current episodes of long duration. Feasibility, acceptance and adherence to the screening of depression were reported by PCPs themselves which adds a recall bias and a social desirability bias into the results, so an overestimation cannot be ruled out. It should be mentioned, that for a small percentage of patients, the study visit was the first contact with the participating PCP, so this may have contributed to the reported underrecognition rates of depression. However, the percentage was balanced between both groups and the inclusion of these patients reflects more precisely the clinical reality of PC. The study was powered to find a difference between groups of 15% or greater; smaller differences, that could be considered clinically relevant by some clinicians, were therefore undetectable. This aspect should be taken into account for future research, especially in pragmatic trials were differences may be smaller than in controlled trials. The focus on high-risk patients, based on USPSTF 2002 recommendations, would have contributed to undermine the chances of showing an effect of the intervention, since the recognition of depression in this group could be higher than among all adults. The intervention was provider-dependent, that is, the PCPs had to do the screening. Support from nursing staff might have had a greater chance of yielding positive results. It could be suggested that screening may hardly be effective in depression because of its episodic nature. Nevertheless, we do not consider this as an explanation for our results, because most patients had been suffering from depression for several months and had consulted their PCP several times.

## Conclusions

This pragmatic randomized trial was not able to show that depression screening under everyday clinical practice in the primary health care setting was effective. The negative results in reducing the underrecognition of MDD in PC may be explained by the poor adherence of the PCPs to the guidelines. Our results reflect the difficulties of putting into practice the clinical guidelines based on controlled clinical studies, into everyday clinical practice. To improve the detection, treatment, and outcomes of MDD, we need to develop and evaluate strategies adapted to the settings where they have to be implemented.

## Competing interests

Irene Romera MD, Sireesha Pamulapati, Belen Yruretagoyena PhD and Inmaculada Gilaberte MD PhD are Eli Lilly employees.

## Authors’ contributions

All authors have collaborated on the design of this study and on the interpretation of the data. Dr. Irene Romera has written the first draft of this article and all authors have contributed to its critical review and approved the final version of the manuscript.

## Prior presentations

This study has not been presented previously.

## Pre-publication history

The pre-publication history for this paper can be accessed here:

http://www.biomedcentral.com/1471-244X/13/83/prepub
